# *Leishmania infantum*-specific production of IL-17a in stimulated blood from dogs in different clinical stages of leishmaniosis

**DOI:** 10.1186/s13071-025-07147-6

**Published:** 2025-11-27

**Authors:** Icíar Martínez-Flórez, Marta Baxarias, Laia Solano-Gallego

**Affiliations:** Departament de Medicina i Cirurgia Animals, Facultat de Veterinaria, Universitat Autonoma de Barcelona, 08193 Bellaterra, Spain

**Keywords:** Canine, Cytokine release whole-blood assay interferon-gamma, Immunity, Disease

## Abstract

**Background:**

*Leishmania infantum* infection progression in dogs depends on the interaction between the parasite and the host’s immune response. The adaptive immune response, primarily mediated by T-helper 1 lymphocytes, promotes an effective reaction by increasing cytokines such as interferon-gamma (IFN-γ). In addition, interleukin-17a (IL-17a) plays a role in controlling parasite growth through inducible nitric oxide synthase activation. However, limited data exist on IL-17a production in dogs at different disease stages. This study aimed to evaluate *L. infantum*-specific IL-17a production in blood samples from dogs with varying clinical stages of leishmaniosis and to assess its correlation with disease severity, humoral response, and IFN-γ  concentrations.

**Methods:**

In total, 65 dogs were included; 10 healthy seronegative and 55 sick dogs, classified into three groups according to the LeishVet clinical stages, were studied. IFN-γ and IL-17a concentrations were measured using a sandwich enzyme-linked immunosorbent assay (ELISA) after performing a *L. infantum*-specific cytokine release whole-blood assay following stimulation with soluble *L. infantum* antigen.

**Results:**

No significant differences in IL-17a concentration were observed between healthy and all sick dogs (*P* = 0.77). Dogs in stage I presented higher IL-17a concentrations than dogs in stages II and III. However, the difference was only statistically significant when compared with stage III (*P* = 0.044). Regarding IFN-γ, all sick dogs demonstrated higher concentrations than healthy dogs (*P* = 0.003). Stage I dogs also exhibited higher IFN-γ concentrations compared with healthy dogs (*P* = 0.0002) and with dogs in stage II (*P* = 0.016) and III (*P* = 0.016). Stage II dogs showed higher IFN- γ concentrations than healthy dogs (*P* = 0.03).

All dogs studied presented a positive correlation between IFN-γ and IL-17a concentrations (Spearman’s *r*: 0.54, *P* < 0.0001). Regarding all the sick dogs, a negative correlation was found between IFN-γ concentration and antibody levels (Spearman’s *r*: −0.41, *P* = 0.002), and between IL-17a concentration and antibody levels (Spearman’s *r*: −0.27, *P* = 0.044). There was a positive correlation between IFN-γ and IL-17a concentration (Spearman’s *r*: 0.61, *P* < 0.0001).

**Conclusions:**

This study demonstrates that IL-17a production is increased in mild disease when compared with more advanced clinical stages, acting as a possible “resistance” marker. However, IL-17a seems to be less reliable as a marker when compared with IFN-γ.

**Graphical Abstract:**

**Figure:**
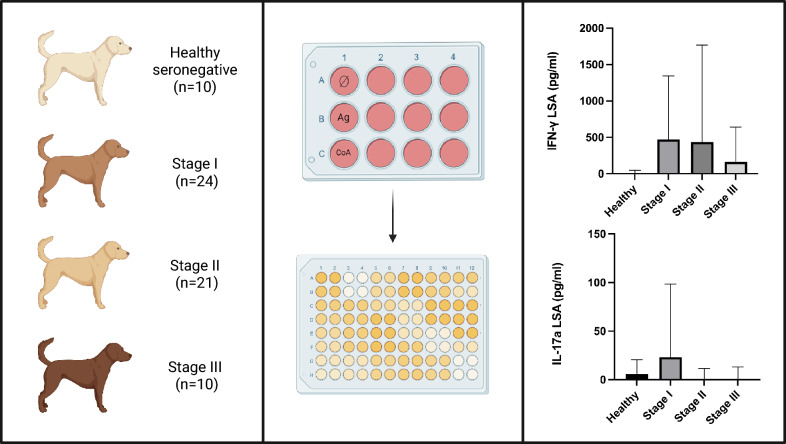

**Supplementary Information:**

The online version contains supplementary material available at 10.1186/s13071-025-07147-6.

## Background

Canine leishmaniosis (CanL) is a zoonotic vector-borne disease caused by the intracellular protozoan *Leishmania infantum* [1]. It is considered a disease of worldwide distribution, but it shows higher prevalence in tropical, subtropical, and Mediterranean countries where it is currently endemic [2].

The infection is caused by the parasite that is transmitted by the bite of female sand flies from the genus *Phlebotomus* and *Lutzomyia* [3]. Although wild animals can be affected by the infection and have been described as potential reservoir [4], dogs are considered the main reservoir host, showing seroprevalence between 3% and 30% in endemic areas [5].

*Leishmania infantum* infection has shown a wide spectrum of clinical disease and severity, from mild cutaneous lesions to life-threatening systemic disease [3, 6]. Canine leishmaniosis is classified into four clinical stages proposed by LeishVet: mild (stage I), moderate (stage II), severe (stage III), or very severe (stage IV) disease on the basis of clinical signs, clinicopathological abnormalities, and measurement of antileishmanial antibodies [3].

The progression of *Leishmania* infection and disease is influenced by the interactions between the parasite and the host’s innate and adaptive immune responses [1, 7, 8]. An adaptive response driven by T-helper 1 lymphocytes (Th1) triggers an effective immune reaction that helps manage the infection by promoting the production of proinflammatory cytokines such as interferon-gamma (IFN-γ), interleukin-2 (IL-2), and tumor necrosis factor-alpha (TNF-α). These cytokines activate macrophages, leading to the production of nitric oxide (NO) and reactive oxygen species (ROS), which work together to eliminate the parasite [9]. The proinflammatory Th1 immune response must be balanced with a regulatory immune response mediated by T regulatory 1 lymphocytes, whose main function is to prevent harmful overactivation of the immune system [10]. Therefore, a strong cell-mediated immune response, involving dendritic cell-primed CD4^+^ (Th1 type) and CD8^+^ T lymphocytes that produce IFN-γ, IL-2, and/or TNF-α, along with the proper activation of T regulatory 1 lymphocytes, is crucial for preventing the spread of the parasite and limiting host tissue damage [11]. In contrast, a T-helper 2 lymphocyte (Th2) response favors a predominantly humoral response and downregulation of cellular immune response, which is not protective and is linked to the progression of the disease [8–12].

The host’s innate and adaptive immune responses to the parasite infection determine the clinical signs and overall outcome of the infection [1, 8]. Consequently, dogs with a strong cellular immune response tend to show resistance to the disease or develop subclinical forms, whereas those with a predominantly humoral response are more likely to experience clinical illness [10, 13]. Besides the cellular differentiation into Th1 or Th2, there is another CD4^+^ T cell subset known as T-helper 17 (Th17) whose function is the production of interleukin-17, among others. Interleukin-17 is a family formed by six cytokines from A to F [14]. Interleukin-17a (IL-17a), produced by various cells, including Th17, natural killer cells, and macrophages [15], contributes to inhibiting parasite growth by activating inducible nitric oxide synthase (iNOS) [16]. In addition, the stimulation of granulocyte–macrophage colony-stimulating factor, interleukin 1, interleukin 6, interleukin 8, TNF-α, and various chemokines amplifies the inflammatory response, among other mechanisms [16, 17]. As a result, IL-17a is linked to resistance against *L. infantum* infection or disease in both humans [16] and dogs [18], working in synergy with IFN-γ. Moreover, this cytokine facilitates the recruitment of neutrophils and other lymphocytes bridging the gap between innate and adaptive immunity. Therefore, it plays a crucial role in the host’s immune defenses against pathogens [15].

As mentioned before, the downregulation of Th1 cells and cytokines such as IFN-γ contributes to the progression of the disease, as well as a decrease in the expression of genes for IL-17a and iNOS [8]. The transcription level of the *IL-17a* gene showed a positive correlation with the mRNA expression of iNOS and IFN-γ. Thus, cytokines associated with Th1 and Th17 responses seem to contribute to limiting parasite growth by activating iNOS and reducing the susceptibility of dogs to disease [17].

However, the function of IL-17a in leishmaniosis is still debated. While some studies propose that IL-17a has a protective effect in leishmaniosis [16, 17], other research suggests that IL-17a might have no link with resistance to *Leishmania* spp. infection in dogs [19] or may even contribute to worsening of the disease in mice with experimental infection of *L. donovani* [20]. This conflicting evidence underscores the need for further research to clarify the specific role of IL-17a in CanL pathogenesis and determine its potential as a prognostic marker. There is limited information available on IL-17a production in dogs with different degrees of disease severity due to *L. infantum* [17]. The aim of this study was to investigate *L. infantum*-specific IL-17a production in stimulated blood from dogs with different clinical stages of leishmaniosis at diagnosis and correlate this finding with disease severity, humoral response, and IFN-γ production.

## Methods

### Dogs and clinical data

A total of 65 dogs were included in this study. Ten healthy seronegative Beagle dogs were purchased from Isoquimen S.L. (Sant Feliu de Codines, Spain) and housed in indoor kennels at Servei de Granges i Camps Experimentals, Facultat de Veterinària, Universitat Autònoma de Barcelona. Ethical approval was obtained by Comissió d’Ètica en l’Experimentació Animal i Humana de la Universitat Autònoma de Barcelona (OH‐CEEA100 i CEEA 4747, July 2019 in the case of the Beagle dogs), (CEAAH 4526, November 2018 for the dogs in stage I), and by Generalitat de Catalunya (10649, June 2021, for the Beagles and FUE-2018-00944112 i ID KSHYD6LVR, April 2019, for the dogs in stage I).The remaining 55 sick dogs were patients at veterinary clinics whose owners provided a signed consent for physical examinations and blood sample collection from their pets. Study authorization was obtained from the Spanish authority, Agencia Española de Medicamentos y Productos Sanitarios (AEMPS) with authorization no. 008/EPA-2383ESP. No dogs were receiving anti-*Leishmania* or anti-inflammatory or immunomodulatory treatment at the time of sample collection.Dogs were divided into four groups: one consisting of healthy seronegative dogs, and the remaining sick dogs were classified according to the LeishVet clinical stages (Table 1) [21]. Group 1 (*n* = 10): healthy seronegative Beagle dogs with no clinical signs nor hematological or biochemical abnormalities; group 2 (*n* = 24): dogs with mild leishmaniosis (LeishVet stage I) presenting with papular dermatitis as the sole clinical sign [22] (diagnosed by intralesional cytology), characterized by the absence of laboratory abnormalities and negative or positive antibody levels; group 3 (*n* = 21): dogs with moderate disease (LeishVet stage II), including the characteristic clinical signs (diffuse clinical manifestations, such as extensive skin lesions, widespread lymph node enlargement, or weight loss among others), hematological and serum biochemical abnormalities with normal creatinine values (< 1.4 mg/dL),and positive antibody levels; and group 4 (*n* = 10): dogs with severe disease (LeishVet stage III), including the characteristic clinical signs and hematological and biochemical abnormalities (including creatinine values of 1.4–2.8 mg/dL or urinary protein/creatinine ratio [UPC] > 1 for stage III and presenting with positive antibody levels [3].

**Table 1:** *Leishmania* antibody.IFN-γ and IL-17a concentrations of dogs according to clinical status and staging

Clinical status and staging	Number of IL-17a producers (%)	IL-17a (pg/mL), median (IQR)	Number of IFN-γ producers (%)	IFN-γ (pg/mL), median (IQR)	Number of IL-17a and IFN-γ producers (%)	*Leishmania* antibody concentrations (EU), median (IQR)
Healthy seronegative (*n* = 10)	0	5.8 (0.8–17.5)	0	0 (0–0)	0	5.8 (4.8–6.5)
Papular dermatitis: stage I (*n* = 24)	9 (37.5)	23.2 (0–135.5)	12 (50)	127.9 (10.2–685.1)	8 (33.3)	21.4 (6.6–66.4)
Stage II (*n* = 21)	2 (9.5)	0 (0–17.3)	4 (19)	9 (0–28.9)	2 (9.5)	2187 (1691–4438)
Stage III (*n* = 10)	1 (10)	0 (0–3.3)	1 (10)	0 (0–49)	1 (10)	2679 (1914–5833)
Total sick dogs (*n* = 55)	12 (21.8)	0 (0–36.3)	17 (31)	20 (0–236.5)	11 (20)	409.4 (25.8–3041)
Total (*n* = 65)	12 (18.5)	2.8 (0–33.7)	17 (26.2)	9.7 (0–140.1)	11 (17)	100.9 (7.4–21.11)

*EU* enzyme-linked immunosorbent assay units, *IFN-γ* interferon-gamma (IFN-γ producers ≥ 110 pg/mL), *IL-17a* interleukin-17a (IL-17a producers ≥ 272 pg/mL), *IQR* interquartile range

A complete physical examination, complete blood count, biochemistry, and in-house enzyme-linked immunosorbent assay (ELISA) to determine serum *L. infantum* antibody levels were carried out for all dogs, and cytologic evaluation of papular dermatitis was performed in stage I to confirm diagnosis. IFN-γ and IL-17a concentrations were measured using a sandwich ELISA after performing an *L. infantum*-specific cytokine release whole-blood assay following stimulation with soluble *L. infantum* antigen [22].

### Blood collection

Blood samples were obtained via jugular or cephalic venipuncture and collected in ethylenediaminetetraacetic acid tubes (BD Vacutainer 2 mL) for hematologic analysis, in heparin tubes (BD Vacutainer 6 mL) for the cytokine release whole-blood assay, and in serum tubes (BD Vacutainer 2 mL) for antileishmanial antibody quantification.

### Enzyme-linked immunosorbent assay for specific *L. infantum* antibody detection

Antibody levels against *L. infantum* antigen were measured using an in-house enzyme-linked immunosorbent assay (ELISA), following methods described in previous studies [1].

Briefly, dog serum samples were diluted 1:800 in phosphate-buffered saline (PBS) with Tween 20 and 1% dry milk and incubated at 37 °C for 1 h on plates coated overnight with sonicated promastigotes of *L. infantum* (MHOM/MON-1/LEM-75) at a concentration of 20 µg/mL [23]. Plates were then washed three times with PBS–Tween and PBS, followed by the addition of horseradish peroxidase-conjugated Protein A (diluted 1:30,000; Merck KGaA, Darmstadt, Germany). After a 1-h incubation at 37 °C and further washes, plates were treated with *o*-phenylenediamine and substrate buffer (SIGMAFAST OPD; Merck KGaA). The reaction was stopped using 5 M H_2_SO_4_, and absorbance was measured at 492 nm with a spectrophotometer (MB-580 HEALES; Shenzhen Huisong Technology Development Co., Ltd., Shenzhen, China). Results were quantified in ELISA units (EU) normalized against a positive canine serum calibrator set at 100 EU.

A threshold of 35 EU was established as previously described [22]. Serum samples were classified as negative (< 35 EU), weak positive (35–150 EU), intermediate positive (150–300 EU), or strong positive (≥ 300 EU).

For intermediate- or strong-positive samples, an ELISA with twofold serial dilutions (starting at 1:800 and extending through 7–11 dilutions) was performed. The quantification was expressed in arbitrary EU relative to a calibrator set at 100 EU (optical density [OD] of 1 at 1:800 dilution). The mean OD values from dilutions approximating 1 were used to calculate EU using the formula: (sample OD/calibrator OD) × 100 × dilution factor [24].

### Cytokine release whole-blood assay and determination of canine IFN-γ and IL-17a

Cytokine release whole-blood assay was performed following established protocols [1].

Briefly, the procedure involved incubating heparinized whole blood under three conditions: (i) medium alone (unstimulated), (ii) medium containing *L. infantum*-soluble antigen (LSA) at 10 µg/mL [25], and (iii) medium with the mitogen concanavalin A (ConA) (100 mg, Medicago^®^, Uppsala, Sweden) at 10 µg/mL. LSA was prepared by subjecting cultured *L. infantum* promastigotes (MHOM/MON-1/LEM-75) at a concentration of 1 × 10^9^ cells/mL in PBS to three freeze–thaw cycles. The supernatant was then collected following centrifugation (8000*g*, 20 min, 4 °C) on the basis of the method previously described, with minor modifications [26].

After a 5-day incubation at 37 °C in a 5% CO_2_ environment, the supernatants were separated by centrifugation (300*g*, 10 min), collected, and stored at −80 °C until analysis.

The concentrations of IFN-γ and IL-17a in all samples were measured using a commercially available sandwich ELISA (DuoSet^®^ ELISA, R&D Systems^™^, Abingdon, UK) with a canine IFN-γ and IL-17a antibody. Standard curves for canine IFN-γ and IL-17a were generated using a four-parameter logistic curve-fit in the MyAssays software (http://www.myassays.com/) [3].

Dogs were classified as *L. infantum*-specific IFN-γ producers when concentrations were ≥ 110 pg/mL [25] and considered IL-17a producers when concentrations were ≥ 272 pg/mL, after subtracting the medium alone [22].

To compare, control, and analyze data more precisely, eliminating irrelevant variations and highlighting truly significant relationships, ratios between IFN-γ concentrations after stimulation with LSA and IFN-γ concentrations after stimulation with ConA were performed. They were also performed in the case of IL-17a concentration after stimulation with LSA and ConA.

### Statistical analysis

Statistical analyses were conducted using GraphPad Prism 8.0.1 for Windows software (GraphPad Software, California, USA).

To determine whether the variables (anti-*L. infantum* antibodies, LSA IFN-γ, and LSA IL-17a) followed a normal distribution, a Shapiro–Wilk test was applied. *P* < 0.05 was considered statistically significant.

To compare the medians of quantitative variables (age, anti-*L. infantum* antibodies, LSA IFN-γ and LSA IL-17a, IFN-γ LSA/IFN-γ ConA ratio, and IL-17a LSA/ IL-17a ConA ratio) among different states of infection (healthy seronegative, stage I, stage II, and stage III), a nonparametric Kruskal–Wallis *H* test was conducted, followed by Dunn’s multiple comparisons test. Following a significant Kruskal–Wallis test result, pairwise comparisons were performed using the Mann–Whitney *U* test. In cases where the Kruskal–Wallis test was not statistically significant, but a potential trend was suspected owing to the small sample size and high intragroup variability, direct Mann–Whitney comparisons between selected pairs of groups were performed.

To examine associations between categorical variables (sex, IFN-γ producers versus IFN-γ nonproducers, and IL-17a producers versus IL-17a nonproducers), Fisher’s exact test was utilized. The Spearman correlation coefficient was calculated to assess relationships between cytokine production and *L. infantum*-specific antibody levels. Differences were deemed statistically significant at a level of 5% (*P* < 0.05).

## Results

### Signalment and clinical data

Overall, 37% of dogs were crossbreeds (*n* = 24) and 63.08% were purebred (*n* = 41), of which the most represented breeds were Beagle (*n* = 10; 15.38%) belonging to the healthy seronegative group and Belgian Malinois (*n* = 4; 6.15%) belonging to the stage I group.

The median age of healthy seronegative dogs was 14.5 months, with a range between 13 and 19 months; 6 months (3–84 months) in the case of stage I, 60 months (12–96 months) for the stage II group, and 48 months (11–120 months) for the stage III group. The total median age was 19 months (3–120 months), with the median age of the sick dogs being 24 months. Significant differences were observed when comparing the age of all four groups (Kruskal–Wallis *H* test, *H* = 41.34; *df* = 3; *P* < 0.0001). Dogs in stage I were significantly younger than dogs in stage II (Mann–Whitney *U* test, *U* = 26; *Z* = 5.95; *P* < 0.0001) and dogs in stage III (Mann–Whitney *U* test, *U* = 12; *Z* = 4.44; *P* < 0.0001).

Regarding sex, the healthy seronegative group had 5 males and 5 females; stage I, 15 males and 9 females; stage II, 10 males and 11 females; and stage III, 9 males and 1 female. The total amount of males was 39 males and 26 females of which 34 males and 21 females belonged to the sick group. No significant differences were found when comparing sex among groups (Fisher’s exact test, *P* = 0.13; odds ratio [OR] = 0.62, 95% confidence interval [CI] 0.16–2.39).

### *Leishmania infantum*-specific antibody levels

**Fig. 1:**
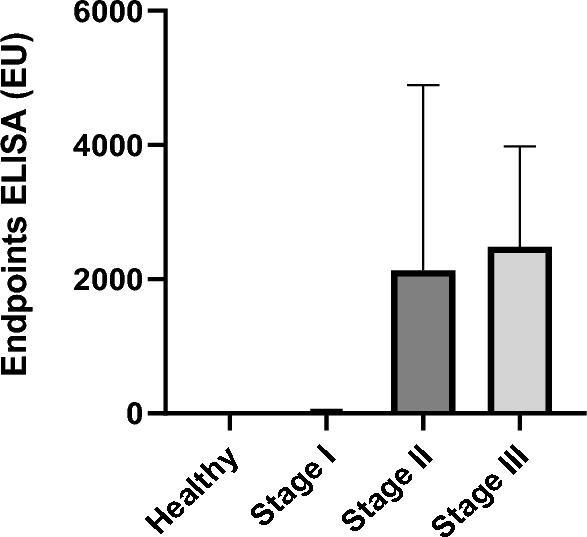
*Leishmania infantum*-specific antibody levels in different *L. infantum* states of infection. Healthy versus stage I, *P* = 0.0036; healthy versus stage II, *P* < 0.0001; healthy versus stage III, *P* < 0.0001; stage I versus stage II, *P* < 0.0001; stage I versus stage III, *P* = 0.003; stage II versus stage III, *P* = 0.42. *EU* ELISA units.

The results of *L. infantum*-specific antibody levels are presented in Table 1 and Fig. 1.

All healthy control dogs were confirmed seronegative (*n* = 10; 5.8 EU, range: 4.4–7.7). Among stage I group (*n* = 24; 21.4 EU, range: 2.8–227.1), 75% of dogs were classified as negative, 21% were classified as weak positive, and 4% were intermediate positive. In the case of stage II group (*n* = 21; 2143 EU, range: 87.2–9782), all dogs were seroreactive, with 9.5% of dogs being considered as weak positive, 42.9% as intermediate positive, and 47.6% as strong positive. Among the stage III group (*n* = 10; 2481 EU, range: 270.8–11114), 10% of dogs were classified as weak positive, 40% intermediate positive, and 50% strong positive.

Significant differences in *L. infantum*-specific antibody levels were observed among the four groups (Kruskal–Wallis *H* test, *H* = 48.94; *df* = 3; *P* < 0.0001). *Leishmania infantum*-specific antibody levels were statistically significantly lower when comparing healthy dogs to sick dogs (Mann–Whitney *U* test, *U* = 45; *Z* = −4.19; *P* < 0.0001). The healthy seronegative group showed significantly lower values when compared with stage I (Mann–Whitney *U* test, *U* = 45; *Z* = −2.85; *P* = 0.004). In addition, the healthy seronegative group showed significantly lower antibody levels when compared with dogs in stage II (Mann–Whitney *U* test, *U* = 0; *Z* = −4.45; *P* < 0.0001) and stage III (Mann–Whitney *U* test, *U* = 0; *Z* = −3.81; *P* < 0.0001). Antibody levels were significantly lower when comparing stage I to stage II (Mann–Whitney *U* test, *U* = 3; *Z* = −5.67; *P* < 0.0001) and stage III (Mann–Whitney *U* test, *U* = 0; *Z* = −4.55; *P* < 0.0001). However, as expected, no statistically significant differences were found when comparing stage II and stage III values (Mann–Whitney *U* test, *U* = 85; *Z* = −0.85; *P* = 0.42) (Fig. 1).

### IFN-γ concentration

**Fig. 2:**
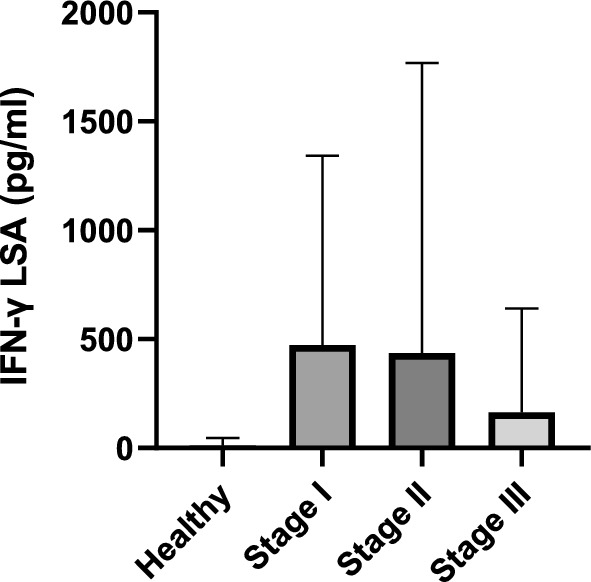
IFN-γ concentrations after stimulation with LSA in different *L. infantum* states of infection. Healthy versus stage I, *P* = 0.0002; healthy versus stage II, *P* = 0.03; healthy versus stage III, *P* = 0.25; stage I versus stage II, *P* = 0.02; stage I versus stage III, *P* = 0.02; stage II versus stage III, *P* = 0.35. *IFN-γ* interferon-gamma, *LSA* *L. infantum*-soluble antigen.

**Fig. 3:**
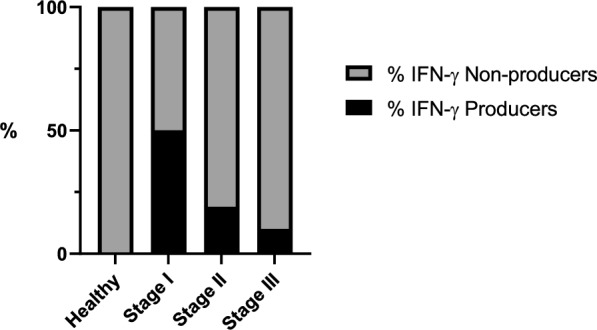
Median concentration with 95% CI after LSA stimulation in IFN-γ producers and nonproducer dogs. *IFN-γ* interferon-gamma, *LSA* *L. infantum*-soluble antigen

The results of the IFN-γ concentration are presented in Table 1 and Figs. 2 and 3.

Significant differences were observed when comparing IFN-γ concentrations among the four groups (Kruskal–Wallis *H* test, *H* = 16.63; *df* = 3; *P* = 0.0008).

Sick dogs (median: 20 pg/mL, range: 0–5691) demonstrated higher IFN-γ concentrations after stimulation with LSA than healthy seronegative dogs (Mann–Whitney *U* test, *U* = 124.5; *Z* = −2.74; *P* = 0.003) when compared as a group. No significant differences were found when comparing IFN-γ concentrations of sick dogs after stimulation with ConA to healthy dogs (Mann–Whitney *U* test, *U* = 168; *Z* = −1.69; *P* = 0.08). Stage I dogs (median: 127.9 pg/mL, range: 0–3998) also exhibited higher IFN-γ concentrations compared with healthy dogs (median: 0 pg/mL, range: 0–109.5) (Mann–Whitney *U* test, *U* = 30; *Z* = −3.4; *P* = 0.0002) and with dogs in stage II (median: 9 pg/mL, range: 0–5691) (Mann–Whitney *U* test, *U* = 148; *Z* = 2.37; *P* = 0.016) and III (median: 0 pg/mL, range: 0–1519) (Mann–Whitney *U* test, *U* = 58; *Z* = 2.34; *P* = 0.016). Stage II dogs showed also higher concentrations than healthy dogs (Mann–Whitney *U* test, *U* = 58.5; *Z* = 1.96; *P* = 0.03). No significant differences were found when comparing dogs belonging to stage III and healthy seronegative dogs (Mann–Whitney *U* test, *U* = 36; *Z* = 1.06; *P* = 0.25) and stage II (Mann–Whitney *U* test, *U* = 119; *Z* = 0.59; *P* = 0.53), respectively (Fig. 2).

Regarding the healthy seronegative group, all dogs (10/10) were classified as IFN-γ nonproducers. In total, 12 dogs out of 24 (50%) from stage I were considered IFN-γ producers (≥ 110 pg/mL). Among the stage II group, 4 dogs out of 21 (19%) were classified as IFN-γ producers. In stage III, only one dog out of ten (10%) was identified as IFN-γ producer (Fig. 3). Statistically significant differences were found when comparing the proportions of IFN-γ producers versus IFN-γ nonproducers in the different groups (Fisherʼs exact test, *P* = 0.007; OR = 0.10, 95% CI 0.87–1.89).

Concerning the IFN-γ LSA/IFN-γ ConA ratios, significant differences were observed when comparing all four groups (Kruskal–Wallis *H* test, *H* = 22.36; *df* = 3; *P* < 0.0001).

There were significant differences between dogs in stage I when comparing with healthy seronegative dogs (Mann–Whitney *U* test, *U* = 28; *Z* = −3.48; *P* = 0.0001), dogs in stage II (Mann–Whitney *U* test, *U* = 129; *Z* = 2.73; *P* = 0.004) and dogs in stage III (Mann–Whitney *U* test, *U* = 33; *Z* = 3.44; *P* = 0.0004). Statistically significant differences were also found between dogs in stage II when compared with healthy seronegative dogs (Mann–Whitney *U* test, *U* = 56.50; *Z* = 1.84; *P* = 0.023). Dogs in stage III did not show differences when compared with stage II (Mann–Whitney *U* test, *U* = 75.50; *Z* = 1.25; *P* = 0.2) and healthy dogs (Mann–Whitney *U* test, *U* = 41.50; *Z* = 0.51; *P* = 0.58).

### IL-17a concentration

**Fig. 4:**
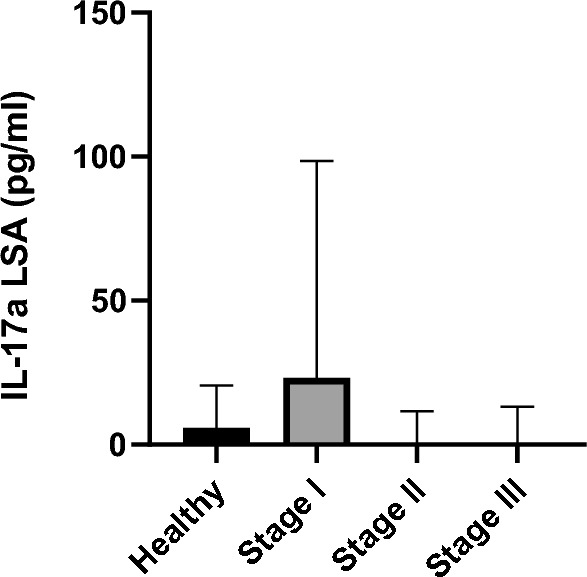
IL-17a concentrations after stimulation with LSA in different *L. infantum* states of infection. Healthy versus stage I, *P* = 0.28; healthy versus stage II, *P* = 0.29; healthy versus stage III, *P* = 0.03; stage I versus stage II, *P* = 0.07; stage I versus stage III, *P* = 0.04; stage II versus stage III, *P* = 0.35. *IL-17a* interleukin-17a, *LSA* *L. infantum*-soluble antigen.

**Fig. 5:**
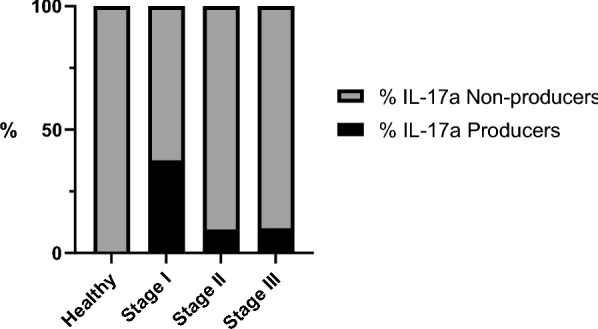
Median concentration with 95% CI after LSA stimulation in IL-17a producers and nonproducer dogs. *IL-17a* interleukin-17a, *LSA* *L. infantum*-soluble antigen

The results of the IL-17a concentration are presented in Table 1 and Figs. 4 and 5.

No significant differences were observed when comparing IL-17a concentrations among the four groups (Kruskal–Wallis *H* test, *H* = 6.7; *df* = 3; *P* = 0.08).

No significant differences in IL-17a concentration after stimulation with LSA were observed between healthy dogs (median: 5.8 pg/mL, range: 0–37.2) and all sick dogs (median: 6.5 pg/mL, range: 0–7679) when compared as a group (Mann–Whitney *U* test, *U* = 461; *Z* = −7.91; *P* = 0.77). No significant differences were found when comparing IL-17a concentrations of sick dogs after stimulation with ConA to healthy dogs (Mann–Whitney *U* test, *U* = 152; *Z* = −26.52; *P* = 0.06). No differences were found when comparing healthy seronegative dogs (median: 5.8 pg/mL, range: 0–37.2) to stage I (Mann–Whitney *U* test, *U* = 91; *Z* = 0.69; *P* = 0.28) and stage II (Mann–Whitney *U* test, *U* = 86; *Z* = 0.68; *P* = 0.29). IL-17a concentrations of healthy seronegative dogs were significantly different from IL-17a concentrations of dogs in stage III (Mann–Whitney *U* test, *U* = 24; *Z* = 1.45; *P* = 0.03). Dogs in stage I (median: 23.2 pg/mL, range: 0–1933) presented higher IL-17a concentrations than dogs in stages II (median: 0 pg/mL, range: 0–6548) and III (median: 0 pg/mL, range: 0–7679); however, the difference was only statistically significant when stage I was compared with stage III (Mann–Whitney *U* test, *U* = 70; *Z* = 2.4; *P* = 0.044). Significant difference was not found comparing dogs in stage II and stage III (Mann–Whitney *U* test, *U* = 81; *Z* = 0.99; *P* = 0.35) (Fig. 4).

Considering the healthy seronegative group, all dogs (10/10) were classified as IL-17a nonproducers. In total, 9 dogs out of 24 (37.5%) from stage I were considered IL-17a producers (≥ 272 pg/mL). Among the stage II group, 2 dogs out of 21 (9.5%) were classified as IL-17a producers. In stage III, only one dog out of ten (10%) was identified as an IL-17a producer (Fig. 5). Significant differences were found when comparing the proportions of IL-17a producers versus IL-17a nonproducers in the different stages (Fisherʼs exact test, *P* = 0.03; OR = 0.17, 95% CI 0.01–2.82).

Regarding the IL-17a LSA/ IL-17a ConA ratios, significant differences were observed when comparing all four groups (Kruskal–Wallis *H* test, *H* = 11.36; *df* = 3; *P* = 0.009).

Statistically significant differences were shown between dogs in stage III and healthy seronegative dogs (Mann–Whitney *U* test, *U* = 14; *Z* = 2.2; *P* = 0.003) and dogs in stage I (Mann–Whitney *U* test, *U* = 49.50; *Z* = 3.22; *P* = 0.004). Statistically significant differences were not found when comparing dogs in stage I with dogs in stage II (Mann–Whitney *U* test, *U* = 181; *Z* = 1.93; *P* = 0.09) and with healthy dogs (Mann–Whitney *U* test, *U* = 88; *Z* = 0.6; *P* = 0.23). In the same way, dogs belonging to stage II did not show differences with stage III dogs (Mann–Whitney *U* test, *U* = 69; *Z* = 1.65; *P* = 0.07) nor with healthy dogs (Mann–Whitney *U* test, *U* = 77; *Z* = 0.91; *P* = 0.22).

Concerning both IFN-γ and IL-17a producers, the healthy seronegative group (all dogs [10/10]) were classified as IFN-γ and IL-17a nonproducers. In addition, 8 dogs out of 24 (33.3%) from stage I were considered IFN-γ and IL-17a producers. Among the stage II group, 2 dogs out of 21 (9.5%) were classified as IFN-γ and IL-17a producers. In stage III, only one dog out of ten (10%) was identified as an IFN-γ and IL-17a producer.

### Correlation between studied parameters

**Fig. 6:**
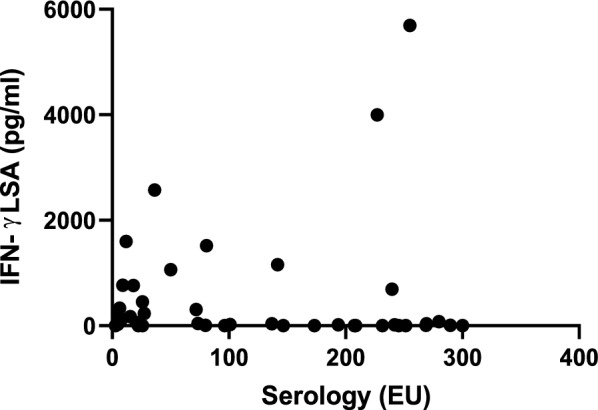
Negative correlation between the IFN-γ concentrations and the *L. infantum*-specific antibody levels in all sick dogs studied. (Spearman’s correlation coefficient, *r*: −0.41, *P* = 0.002). *IFN-γ* interferon-gamma, *LSA* *L. infantum*-soluble antigen

**Fig. 7:**
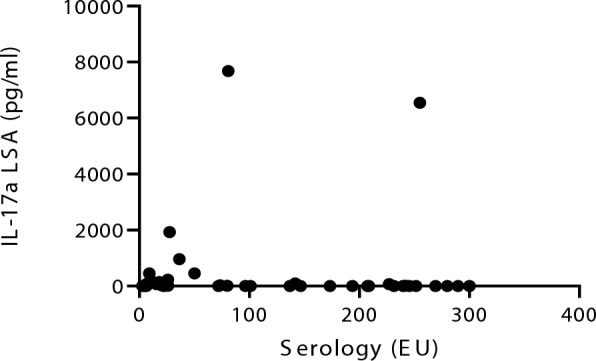
Negative correlation between the IL-17a concentrations and the *L. infantum*-specific antibody levels in all sick dogs studied. (Spearman’s correlation coefficient, *r*: −0.27, *P* = 0.044). *IL-17a* interleukin-17a, *LSA* *L. infantum*-soluble antigen

**Fig. 8:**
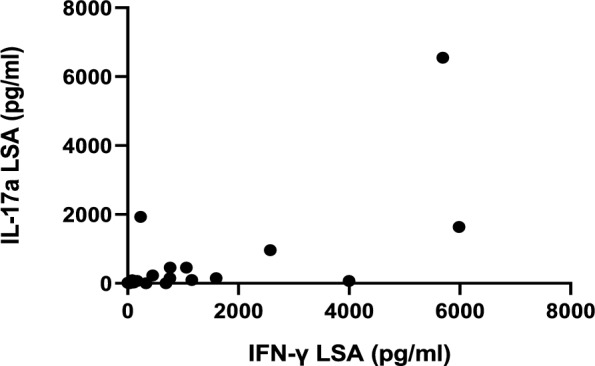
Positive correlation between the IL-17a and IFN-γ concentrations in all sick dogs studied. (Spearman’s correlation coefficient, *r*: 0.61, *P* < 0.0001). *IFN-γ* interferon-gamma, *IL-17a* interleukin-17a, *LSA* *L. infantum*-soluble antigen

All studied dogs presented a positive correlation between IFN-γ and IL-17a concentrations (Spearman’s correlation coefficient, *r*: 0.54, *P* < 0.0001). Regarding all the sick dogs, a negative correlation was found between IFN-γ concentration and antibody levels (Spearman’s correlation coefficient, *r*: −0.41, *P* = 0.002), and between IL-17a concentration and antibody levels (Spearman’s correlation coefficient, *r*: −0.27, *P* = 0.044) (Figs. 6 and 7, respectively). However, there was a positive correlation between IFN-γ and IL-17a concentrations (Spearman’s correlation coefficient, *r*: 0.61, *P* < 0.0001) (Fig. 8).

## Discussion

This is the first study to evaluate *L. infantum*-specific IL-17a production in stimulated blood in dogs with different clinical stages of leishmaniosis at diagnosis and correlate this information with disease severity, humoral response, and IFN-γ production. The results obtained in this study showed that dogs belonging to stage I demonstrated higher IL-17a concentrations than dogs with moderate-to-severe disease. In addition, stage I group showed a higher percentage of dogs considered as IL-17a producers when comparing with healthy seronegative, stage II, and stage III groups.

In this study, all dogs in stage I showed papular dermatitis as the only clinical sign, which is considered a benign cutaneous manifestation that often shows self-healing of the skin lesions, meaning that these dogs demonstrated mild leishmaniosis [27]. Papular dermatitis is associated with effective Th1-dominant parasite-specific cellular immunity and a minimal humoral immune response. In addition, it is typically characterized by the absence of laboratory abnormalities and systemic clinical signs [22, 27].

The higher IL-17a levels in stage I dogs could reflect an effective early immune response limiting disease progression. This observation aligns with previous research underscoring the potential protective role of IL-17a in controlling *Leishmania* infection [16, 28]. IL-17a exerts potent proinflammatory effects by inducing the production of NO, granulocyte–macrophage colony-stimulating factor, interleukin-1, interleukin-6, interleukin-8, TNF-α, and various chemokines, which collectively enhance macrophage activation and pathogen clearance [29, 30]. The production of these inflammatory mediators suggests that IL-17a plays a role in intracellular parasite infections, as evidenced by studies on *Trypanosoma cruzi* infections in humans [16, 31].

In addition, IL-17a could have a significant role in the recruitment of neutrophils, which are pivotal in mediating pathogen elimination through mechanisms such as phagocytosis, ROS production, and neutrophil extracellular trap formation [29, 30, 32]. Evidence suggests that the IL-17-mediated neutrophilic influx is critical for controlling parasite replication and fostering a Th1-biased immune environment in humans [28]. This is also supported by studies demonstrating that IL-17-deficient murine models exhibit impaired Th1 responses and increased parasite burdens, underscoring the protective role of this cytokine [33]. Thus, higher IL-17a could contribute to the self-limiting characteristics of patients with papular dermatitis (stage I group) by potentially promoting Th1 immunity and a rapid neutrophil recruitment to infection sites, facilitating parasite clearance before extensive dissemination occurs, similar to what has been described in humans [34].

The decline in IL-17a levels observed in more advanced stages (stages II and III) may signify an ineffective immune response. In humans, the absence of IL-17a signaling leads to the generation of a T-reg/IL-10-dominated response and an impaired Th1 profile, resulting in parasite growth [16, 32]. Alternatively, low IL-17a levels in these patients could reflect immune exhaustion. T cell exhaustion refers to the dysfunction of T cells resulting from prolonged exposure to antigens, and it is characterized by the heightened expression of inhibitory receptors such as programmed death-1 and cytotoxic T-lymphocyte-associated antigen 4 [35, 36]. T cell exhaustion in canine leishmaniosis has been reported and associated with a reduction in other protective cytokines such as IFN- γ [37]. In these patients, reduced IL-17a likely parallels impaired Th1/Th17 responses, potentially leading to inadequate macrophage activation and uncontrolled parasite replication, contributing to disease exacerbation.

In human medicine, IL-17a has attracted considerable attention for its proinflammatory role in autoimmune diseases. When dysregulated, IL-17a contributes to the pathogenesis of diverse inflammatory and autoimmune conditions, such as in psoriasis, psoriatic arthritis, ankylosing spondylitis, and multiple sclerosis [38, 39]. In this context, it has shown to promote keratinocyte hyperproliferation, chronic neutrophilic inflammation, and tissue destruction [38]. At the same time, IL-17a is crucial for mucosal protection against extracellular pathogens through the recruitment of neutrophils, induction of antimicrobial peptides, modulation of T cell differentiation, and maintenance of epithelial barrier integrity [39, 40]. Furthermore, IL-17a also participates in tissue repair and can influence cancer progression by supporting angiogenesis and tumor growth, though context-dependent antitumor roles have also been described [38, 40]. Thus, the IL-17a cytokine family plays a dual role in immunity, being indispensable for host defense while also contributing to chronic inflammation and autoimmunity. Taken together, our findings indicate that, while dysregulated IL-17a contributes to pathology in autoimmune diseases such as psoriasis or arthritis in humans, in canine leishmaniosis, IL-17a may serve a protective role in promoting an effective immune response.

As mentioned above, *Leishmania* infection and disease progression depend on the dynamic interplay between the parasite and the host’s innate and adaptive immune responses [1, 8, 41]. A Th1-mediated adaptive immune response plays a pivotal role in infection control by driving the production of cytokines such as IFN-γ, IL-2, and TNF-α. These cytokines stimulate macrophages to produce NO and ROS, leading to the elimination of the parasite [9]. Among these, IFN-γ stands out as a potent immunoregulatory cytokine that not only orchestrates the adaptive immune response but also enhances neutrophil activity [42, 43].The production of *L. infantum*-specific IFN-γ in stimulated blood is a key indicator of a strong Th1-mediated immune response and has been strongly linked to disease control and a resistant phenotype across multiple species. Consistent with this, elevated IFN-γ levels have been observed in dogs with mild, self-limiting clinical leishmaniosis, underscoring its association with infection control and milder clinical outcomes during *Leishmania* infection [8, 10, 44, 45].

In the present study, 50% (12/24) of dogs in stage I were identified as IFN-γ producers. In contrast, only 19% of dogs in stage II and 10% in stage III were classified as IFN-γ producers. Regarding the IFN-γ median concentrations, dogs in stage I showed higher levels than dogs belonging to stage II and stage III as well as healthy seronegative dogs. These results align with previous studies that utilized IFN-γ release whole-blood assays in dogs with stage I and papular dermatitis. One study reported that 58% of dogs were classified as IFN-γ producers, with dogs in stage I (25.7%) and IIa (48.5%) exhibiting higher IFN-γ production compared with those in stages IIb (8.5%), III (14.2%), and IV (2.8%) [1]. Similarly, another study found that 15 out of 19 dogs in stage I (79%) were IFN-γ producers, whereas only 6 of 15 dogs (40%) in stages II–III were classified as such [7]. In addition, another study reported that 78% of IFN-γ-producing dogs were classified as stage I or IIa, while 43% of IFN-γ nonproducers were in stage IIb. Notably, none of the stage I dogs were classified as IFN-γ nonproducers [45]. In our study, most dogs in stages II and III were categorized as nonproducers of IFN-γ. As observed in previous studies, dogs in the more advanced clinical stages exhibited reduced IFN-γ levels, indicating a potentially less effective immune response that deviates from either a Th1-driven phenotype or T cell exhaustion [37, 46].

Our study identified a positive correlation between IL-17 and IFN-γ concentrations in dogs naturally infected with *L. infantum*, highlighting the potential interplay of these cytokines in the host immune response. Notably, in different murine and in vitro studies, IL-17 was reported to synergize with IFN-γ to potentiate NO production in infected macrophages, a mechanism critical for effective antileishmanial activity [16, 33, 47]. In addition, previous studies performed in murine models for bacterial pneumonia have demonstrated that IL-17 can indirectly enhance IFN-γ production by promoting the recruitment and activation of neutrophils, which, in turn, secrete IL-12, a critical cytokine for inducing IFN-γ release [48–50]. Such synergy between IL-17 and IFN-γ underscores their complementary roles in sustaining a robust Th1-mediated immune response, essential for controlling intracellular pathogens such as *L. infantum*. Our results align with these findings and suggest that IL-17 may play a role in sustaining the IFN-γ-mediated immune response in dogs naturally infected with *L. infantum*. While the role of IL-17 in leishmaniosis is complex and may vary depending on the species and clinical context, its association with increased IFN-γ production in our study supports their synergy and contributes to maintaining an inflammatory microenvironment conducive to parasite control. Further investigation is warranted to elucidate the specific mechanisms underlying IL-17 and IFN-γ interplay in CanL and their implications for disease progression and clinical outcomes.

Considering serological status, dogs in stage II showed the highest *L. infantum*-specific antibody levels, since 90.5% of individuals were considered as intermediate and strong positive. Dogs belonging to stage III also demonstrated high antibody levels as expected. In contrast, most of the dogs in stage I were classified as seronegative (75%). Moreover, all sick dogs demonstrated a negative correlation between IFN-γ concentrations and *L. infantum*-specific antibody levels [51]. This kind of relationship may be explained by the role of IFN-γ as an indicator of a strong Th1 response, which is predominantly cellular rather than humoral. In contrast, elevated antibody levels are associated with a vigorous humoral immune response, which is often linked to other cytokines such as IL-4, IL-10, and TGF-β [8, 9, 12]. A negative correlation was also found between IL-17a concentration and *L. infantum*-specific antibody levels, which may reinforce the hypothesis that IL-17a is a marker of resistance to the disease.

One of the main limitations of this study was the small and uneven sample sizes across the different groups. Furthermore, we were unable to include dogs in stage IV of the disease, limiting the generalizability of our findings to the most advanced stage of leishmaniosis. In addition, healthy seropositive dogs were not included. Despite these limitations, the results provide valuable preliminary data that can guide the design of future studies with larger and more balanced cohorts and with different states of infection.

## Conclusions

The results of this study demonstrated that IL-17a production is increased in mild disease (stage I) when compared with more advanced clinical stages (II and III). Despite that IL-17a seems to be less reliable as a marker when compared with IFN-γ, these findings highlight the potential role of IL-17a as a biomarker for effective immune response in CanL.

## Supplementary Information

Additional file 1.

## Data Availability

All data supporting the main conclusions are available in the manuscript and in Additional File 1 (Dataset S1: signalment, clinical data, and immunological results).

## Abbreviations

CanL: Canine leishmaniosis
ConA: Concanavalin A
ELISA: Enzyme-linked immunosorbent assay
EU: ELISA units
iNOS: Inducible nitric oxide synthase
IFN-γ: Interferon-gamma
IL-2: Interleukin-2
IL-4: Interleukin-4
IL-10: Interleukin-10
IL-17a: Interleukin-17a
LSA: *Leishmania infantum*-soluble antigen
NO: Nitric oxide
OD: Optical density
PBS: Phosphate-buffered saline
ROS: Reactive oxygen species
Th1: T-helper 1 lymphocytes
Th2: T-helper 2 lymphocytes
TGF-β: Transforming growth factor-beta
TNF-α: Tumor necrosis factor-alpha
UPC: Urinary protein/creatinine ratio
